# Seminal Vesicle Calculi as a Cause of Hematospermia and Ejaculatory Pain: A Case Report

**DOI:** 10.7759/cureus.42547

**Published:** 2023-07-27

**Authors:** Vanessa Andrade, João Pina, Fernando Calais, Luís Campos Pinheiro

**Affiliations:** 1 Urology, Centro Hospitalar Universitário de Lisboa Central, Lisboa, PRT

**Keywords:** hematospermia, robotic surgery, seminal vesicle calculi, perineal pain, ejaculatory pain

## Abstract

Seminal vesicle calculi are a rare entity that may present with hematospermia, painful ejaculation, or urinary complaints. We present a case of a 40-year-oldmale with complaints of hematospermia, ejaculatory pain, and perineal discomfort in the last five years. A 7 mm left seminal vesicle calculi were diagnosed by magnetic resonance imaging (MRI), and a laparoscopic robot-assisted vesiculectomy was performed. All the complaints improved completely after treatment.

Seminal vesicle lithiasis should be kept in mind when evaluating patients with hematospermia and ejaculatory pain. Transrectal ultrasound (TRUS) and magnetic resonance imaging are the best radiology techniques to diagnose this kind of lithiasis. Different surgical treatments can be used to treat these calculi, depending on the size and location of the calculi and the surgical experience of the surgeon.

## Introduction

The seminal vesicles are two glands located posteriorly to the bladder and are responsible for producing seminal fluid that provides a nutritional environment to spermatozoa, with contributes to 80% of ejaculate volume [1].

The first case of seminal vesicle calculi was reported in 1928 by White [2]. Seminal vesicle calculi are a rare occurrence that have been diagnosed and reported more frequently in the last years because of the increased awareness about the investigation of hematospermia and ejaculatory pain. The etiology of these calculi is frequently unknown. The investigation of hematospermia using transrectal prostatic ultrasound, which is now widely available, had also an impact on the increasing number of diagnosed cases [1].

We report a case of a 40-year-old male who presented with painful ejaculation, perineal discomfort, and hematospermia diagnosed with seminal vesicle calculi successfully treated by robot-assisted laparoscopic surgery.

## Case presentation

**Figure 1:**
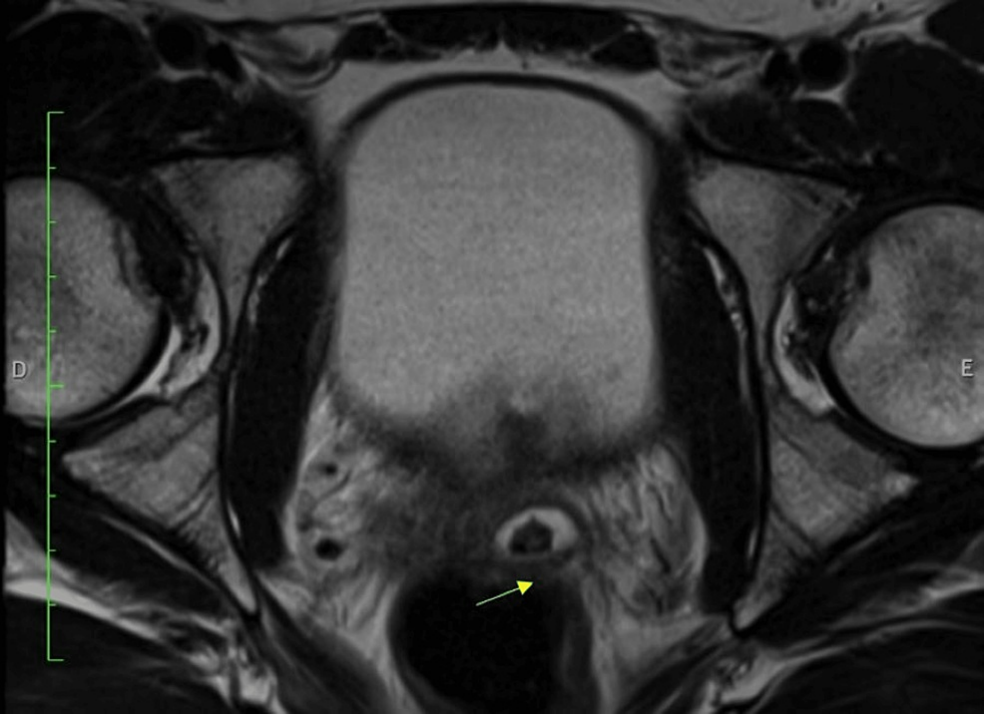
T2 MRI axial view. The arrow shows the left seminal vesicle with the calculi inside. MRI: magnetic resonance imaging

**Figure 2:**
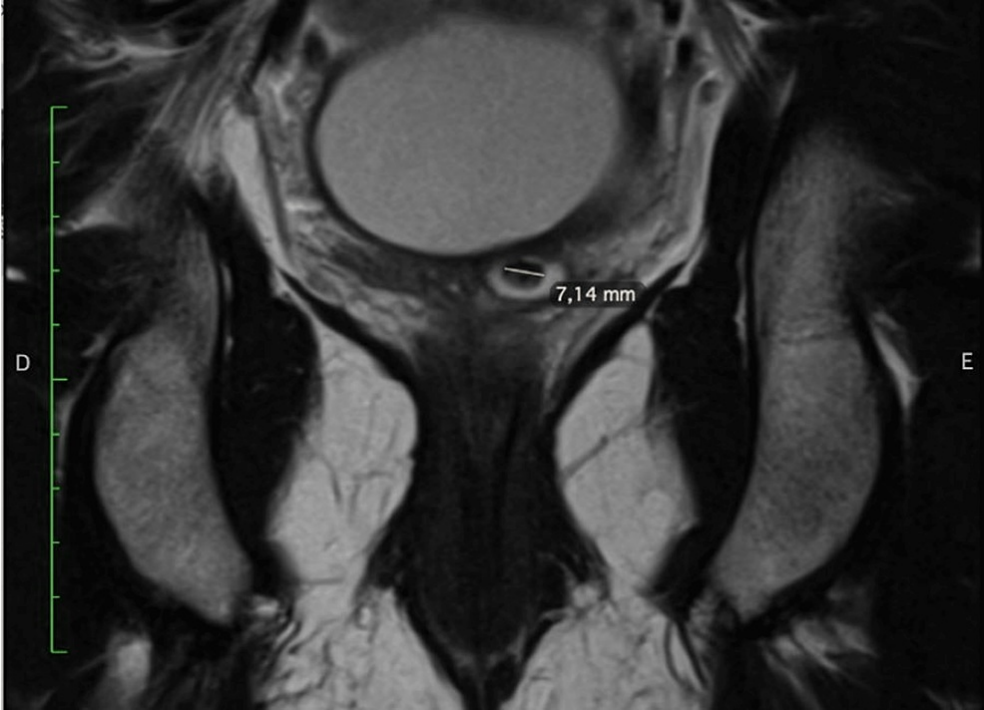
T2 MRI coronal view. MRI: magnetic resonance imaging

We report a case of a 40-year-old male who presented with a medical history of hematospermia, painful ejaculation, and perineal discomfort for five years. He denied any previous medical issues and any urinary complaints such as urgency, frequency, or dysuria. These complaints, specially hematospermia, had a real impact on the sexual life of the patient, who had reduced sexual activity due to it. He was medicated with different antibiotics and anti-inflammatory drugs, without any improvement. Urine culture was negative. General blood analysis was completely innocent. Prostate-specific antigen (PSA) was normal: 0.31 ng/mL. Urethrocystoscopy was performed and showed a normal urethra with a nonobstructive prostate and bladder mucosa with normal characteristics. Transrectal ultrasound (TRUS) showed a 30 cc prostate without any abnormality. Due to persistent complaints, a pelvic magnetic resonance imaging (MRI) was performed, showing asymmetric seminal vesicles, due to the enlarged volume of the left one probably because of hematic content and a possible lithiasic focus in prostatoseminal angle measuring 7 mm (Figures 1, 2).

The patient wanted to do a vasectomy, so fertility issues were not a problem.

**Figure 3:**
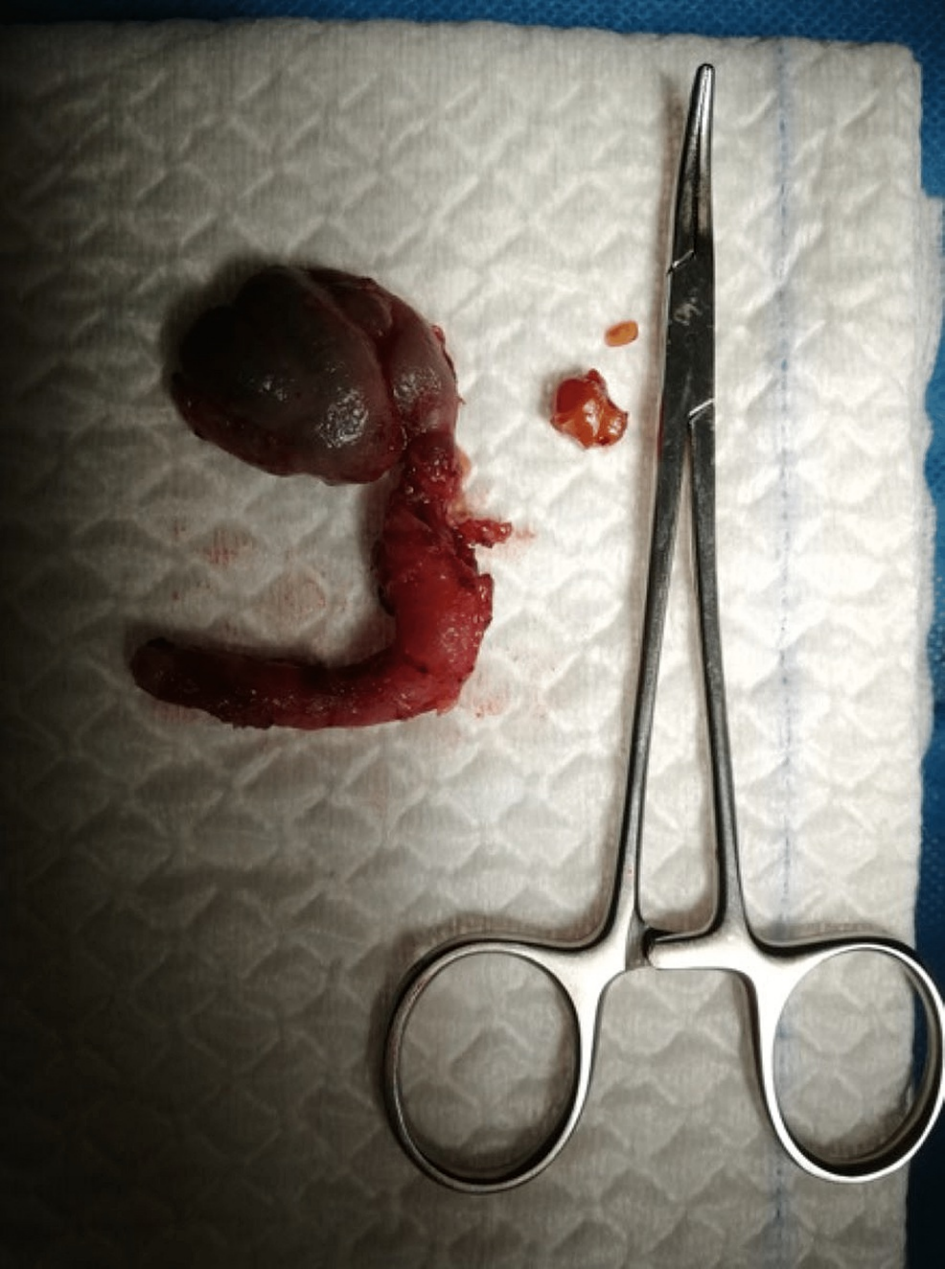
The left seminal vesicle removed and two calculi.

A robot-assisted laparoscopic excision of the left seminal vesicle was performed, due to the lack of experience (and proper material) in our center with endoscopic approach. Four 8 mm trocars were placed in line, one above the umbilicus, two on the left (one with 12 mm and one with 5 mm for suction), and one on the right. Under the direct vision of cul-de-sac, the seminal vesicle, bladder, and rectum were visualized. A transversal incision was made in the retrovesical peritoneum. The seminal vesicles were found, and the left one was separated cautiously from the structures around until the prostatoseminal angle, where the calculi were found. When completely freed, the structure was removed en bloc (Figure 3).

The defect left and the peritoneum were closed with a Vicryl® 2/0 suture (Ethicon, Inc., Cincinnati, OH). The patient only received prophylactic antibiotics during surgical intervention. He was discharged the day after the surgery with painkillers and one-week antibiotic. Six months after the surgery, he showed total remission of the symptoms.

## Discussion

We decided to report this case because of its rarity; as a result, we can alert the clinicians to the existence of this condition that can really affect the quality of life of our patients but can be easily managed after the diagnosis.

Seminal vesicle calculi can present with a wide range of clinical presentations, such as hematospermia and ejaculatory pain, the most common symptoms, or even perineal, abdominal, or testicular pain and urinary complaints such as dysuria, frequency, or urinary tract infections [1]. This ejaculatory duct obstruction can also be a cause of infertility. In our patient, perineal discomfort was one of the main complaints, which can be a really nonspecific complaint. Fertility was not an issue for him, given that the obstruction was unilateral.

The etiology of these calculi is frequently unknown. One cause can be an impaired drainage of the ejaculate due to, for example, a seminal vesicle cyst or Müllerian duct remnants [1]. Corriere suggested that stasis and the lack of proteases in semen could contribute to calculi formation [3]. The impaired drainage of the ejaculate, urine reflux with stasis, and aggregation of debris, combined with urinary tract infections and vesiculitis, can also lead to the formation of calculi [1]. Metabolic disorders that predispose to crystal deposition can be another cause. Different compositions have been described, but a direct correlation with systemic diseases is not clear [4]. Some studies suggest that the formation of these calculi is mainly due to infection [4].

The composition of seminal vesicle calculi can vary widely. There have been calculi reported to be composed of calcium phosphate, calcium oxalate monohydrate, carbonate apatite, struvite, protein, and other serum-like organic matter [4].

Physical examination and imaging studies are necessary for the diagnosis. A digital rectal examination can sometimes identify hard calculi [4]. In our case, a digital rectal examination was performed but could not find any asymmetry or hard calculi, maybe because of its reduced dimensions.

Before the evolution of radiology techniques, plain pelvic X-ray and intravenous urography were used to identify calcifications of the urinary tract, including the seminal vesicles. Of course, the usefulness of these techniques depends if the calculi are radiopaque or not, and some can be missed [5].

Transrectal ultrasound has detected at least 50% of seminal vesicle cases reported in the literature. It can detect seminal and ejaculatory duct dilatation with or without calculi, cysts, and prostatic calcifications [6]. MRI is the safest and most sensitive mode of imaging for the seminal vesicle tract, compared to TRUS or computed tomography (CT), as MRI can detect calculi difficult to detect by other examinations, such as calculi composed mainly of proteinaceous material [7]. Transurethral seminal vesiculoscopy (TRU-SVS) is performed through cystoscopy; then, a catheter is passed into the ejaculatory ducts, and retrograde seminal vesiculography is performed. This procedure can add some information about symptoms, anatomy, and stone burden allowing better surgical planning. However, compared to TRUS, this procedure requires general anesthesia, antibiotic coverage, and surgical experience [1].

The choice of treatment depends not only on the size and location of the calculi but also on the general condition and previous surgeries of the patient. In the past, the treatment of choice for large calculi was open surgery and cystostomy, which carried a lot of morbidity [1]. Transrectal and transperitoneal vesiculotomy and the removal of the calculi were also performed. With the evolution of minimally invasive surgery and endourology instruments, new approaches appeared. Ozgök et al*. *were the first authors to report on the endoscopic treatment of seminal vesicle calculi. They catheterized the utricular orifice and passed a 6.9 F flexible ureteroscope through a guidewire and crushed the calculi with a grasper [2]. Later, Cuda et al. used a 7 F semirigid ureteroscope and a 270 µm holmium laser fiber to destroy the calculi [8]. Modi used a 6.6 F ureteroscope and a pneumatic lithotripter [9]. Transurethral seminal vesiculoscopy (TRU-SVS) is a safe, minimally invasive procedure that can resolve hematospermia, seminal vesiculitis, infertility, and ejaculatory duct obstruction [1]. The calculi can be removed using a grasping forceps or through fragmentation with a holmium laser [10]. Irrigation with antibiotics after the procedure can help in reducing the infection rate [4]. This procedure is a good option for the treatment of small calculi, if the surgeon has enough experience.

The laparoscopic surgery of the seminal vesicles was first described in 1993 by Kavoussi et al. [11]. Nowadays, robotic vesiculotomy or vesiculectomy can also be done. These are the best approaches for large calculi and the treatment of seminal vesicle cysts.

## Conclusions

High suspicion is needed to diagnose seminal vesicle calculi when males have sexual (hematospermia and ejaculatory pain) or urinary complaints. Due to its rarity, the etiology and pathogenesis of the formation of the stone are unclear. Several surgical approaches have been described. The surgeon should choose the best technique regarding the location, size, and surgical experience and skills. When treating the calculi, the rate of success is high, and patients who spent years to understand their complaints usually get asymptomatic after treatment. More investigation is needed about calculi composition and its causes so we can understand better which are the main risk factors and maybe act on them.

## References

[1] Christodoulidou M, Parnham A, Nigam R (2017). Diagnosis and management of symptomatic seminal vesicle calculi. Scand J Urol.

[2] Ozgök Y, Kilciler M, Aydur E, Saglam M, Irkilata HC, Erduran D (2005). Endoscopic seminal vesicle stone removal. Urology.

[3] Corriere JN Jr (1997). Painful ejaculation due to seminal vesicle calculi. J Urol.

[4] Miao C, Liang C, Wang Y (2020). The management and composition of symptomatic seminal vesicle calculi: aetiological analysis and current research. BJU Int.

[5] Li YK (1991). Diagnosis and management of large seminal vesicle stones. Br J Urol.

[6] Razek AA, Elhanbly S, Eldeak A (2010). Transrectal ultrasound in patients with hematospermia. J Ultrasound.

[7] Lencioni R, Ortoris S, Cioni D (1999). Endorectal coil MR imaging findings in hemospermia. MAGMA.

[8] Cuda SP, Brand TC, Thibault GP, Stack RS (2006). Case report: endoscopic laser lithotripsy of seminal-vesicle stones. J Endourol.

[9] Modi PR (2006). Case report: endoscopic management of seminal vesicle stones with cutaneous fistula. J Endourol.

[10] Zaidi S, Gandhi J, Seyam O, Joshi G, Waltzer WC, Smith NL, Khan SA (2019). Etiology, diagnosis, and management of seminal vesicle stones. Curr Urol.

[11] Kavoussi LR, Schuessler WW, Vancaillie TG, Clayman RV (1993). Laparoscopic approach to the seminal vesicles. J Urol.

